# Autologous Platelet-Rich Plasma (PRP) Efficacy on Endometrial Thickness and Infertility: A Single-Centre Experience from Romania

**DOI:** 10.3390/medicina59091532

**Published:** 2023-08-24

**Authors:** Anca Huniadi, Ioana Alexandra Zaha, Petronela Naghi, Liana Stefan, Liliana Sachelarie, Alin Bodog, Erika Szuhai-Bimbo, Codruta Macovei, Mircea Sandor

**Affiliations:** 1 Faculty of Medicine and Pharmacy, University of Oradea, 1St December Square 10, 410073 Oradea, Romania; ancahuniadi@gmail.com (A.H.); lianaantal@gmail.com (L.S.); bszera@gmail.com (E.S.-B.); icmek69@gmail.com (C.M.); drims75@gmail.com (M.S.); 2 Calla—Infertility Diagnostic and Treatment Center, Constantin A. Rosetti Street, 410103 Oradea, Romania; drzahaioana@gmail.com (I.A.Z.); petronelanaghi@gmail.com (P.N.); 3 Pelican Clinical Hospital, Corneliu Coposu Street 2, 410450 Oradea, Romania; 4 Department of Clinical Discipline, Apollonia University, 700511 Iasi, Romania

**Keywords:** fertilization, endometrial thickness, platelet-rich plasma (PRP)

## Abstract

(1) *Background*: During IVF (in vitro fertilization), a proper endometrium thickness is one of the most difficult parameters to achieve and one of the most important prognostic factors of the success rate. One major problem is the high cancelation percentage in frozen embryo transfer cycles. The focus on the adjuvant methods for improving endometrium thickness is an on-going subject of interest. (2) *Methods*: This prospective single-arm self-control study was conducted in an IVF centre in Oradea, Romania. The patients were divided into two groups. The control group included 51 patients with at least one attempt to transfer a good-quality blastocyst, but the endometrial thickness did not surpass 7 mm under standard endometrial preparation protocol with oestradiol and with adjuvant therapy (other than PRP, such as aspirin, vitamin C, and vitamin E), and the study group included the same 51 patients that had the embryo transfer performed under the same standard endometrial preparation protocol with oestradiol preparation protocol and intrauterine PRP infusion. (3) *Results*: In our study, the PRP treatment had a positive impact on the parameters that were followed for the evaluation of the success rate of the embryo transfer procedure. The endometrial thickness (an increase in endometrial thickness by 0.6 mm after PRP treatment with *p* = 0.0001) and the clinical pregnancy rate (having a MD ± SD of 0 ± 0.38 before PRP treatment and with an increase to 0.5 ± 0.1 after the PRP treatment, *p* = 0.0004) were statistically significant (4) *Conclusions*: PRP has a positive effect in promoting endometrial proliferation, improving embryo implantation rate and clinical pregnancy rate for women with thin endometrium.

## 1. Introduction

Endometrium thickness and a good-quality blastocyst are two of the main factors involved in embryo transfer for achieving clinical pregnancy. During IVF (in vitro fertilization), a proper endometrium thickness is one of the most difficult parameters to achieve and one of the most important prognostic factors in the success rate.

Different strategies for managing infertility have been suggested in recent years. However, many embryos fail to implant despite the availability of a variety of assisted reproductive technology (ART) procedures [1,2,3,4,5,6,7,8]. Cytokines, growth factors, prostaglandins, and different binding molecules are secreted in the endometrium during the endometrial receptivity period. The right state of the endometrium is one of the key elements in embryo implantation.

A woman’s endometrium, an inner layer of epithelial tissue that lines the uterus, is a special tissue that goes through countless cycles of development, differentiation, and detachment during her lifetime [1,2]. Morphologically normal endometrium is one of the crucial requirements for the success of assisted reproductive technology (ART) programs. The evidence that is currently available indicates that between 50 and 70 percent of implantation failures are related to poor endometrial receptivity; 7 mm or greater endometrial thickness is ideal for embryo transfer [3,4]. Thin endometrium in assisted reproduction is often defined as endometrial thickness <7 mm or <8 mm [4]. Thin endometrium, or insufficient endometrial thickness, causes low implantation, reproductive failures, and the termination of ART programs. In these circumstances, there is a high risk of early miscarriages, preterm delivery, and low-birth-weight children [5].

For effective implantation, the endometrium must include a variety of growth hormones, cytokines, and lipids that are essential for endometrial and embryonic development. The endometrium develops in response to various factors, including hormone levels [6,7,8].

The thickness of the endometrium was used to establish the maximum distance in the uterine longitudinal plane between the two endometrium–myometrium junction surfaces [8,9]. Thin endometrium is defined as an endometrium under 7 mm on the day of ovulation or HCG (human chorionic gonadotrophin) trigger in fresh cycles. In cases of frozen embryo transfers, we consider a ”thin endometrium” to be an endometrium that does not achieve 7 mm on the day of progesterone supplementation after a standard oestradiol protocol preparation [1,2].

The endometrium is part of the three major factors for a successful implantation: embryo implantation competency, endometrium receptivity, and synchronization of the embryo and endometrium [2,3,4]. Cytokines, growth factors, adhesion molecules, and cytoskeletal proteins, among other substances, participate in and regulate the complicated process of endometrial receptivity [5,6,7,8,9].

Thin endometrium remains a challenge for specialists in the human reproduction field, and the fact that with traditional methods we can hardly achieve the desired thickness—considered by most studies to be at least 7 mm—makes it necessary to introduce new techniques for improving the structure and thickness of the endometrium [3]. Adequate thickness, while crucial, is also difficult to determine. However, pregnancies with an EMT (endometrial thickness) as low as 4 mm have been reported, and studies have consistently shown that patients can achieve a clinical pregnancy with an endometrial thickness of <8 mm, but this is associated with poor placentation and pregnancy complications like pregnancy loss, hypertensive disorders, preterm delivery, and placenta praevia [6,7].

One major problem is the high cancelation percentage in frozen embryo transfer cycles with thin endometrium in different endometrial preparation protocols. The focus on the adjuvant methods for improving endometrium thickness is an on-going subject of interest. 

Along with the administration of sildenafil citrate, pentoxifylline, low-dose aspirin and vitamin E supplementation in attempts to obtain better results in frozen embryo transfer protocols, the infusion of autologous activated platelet-rich plasma (PRP) in the uterine cavity has received attention in recent years [4,5]. 

Initially, PRP was used in surgical fields like dermatology, plastic surgery, and orthopaedics [6,7,8]. 

Animal studies demonstrated a decrease in the expression of inflammatory markers and fibrosis, and increased endometrial proliferation rate, increased expression of proliferative genes, and increased clinical pregnancy rates [9]. 

PRP can be made quickly by centrifuging blood samples to create plasma that is highly concentrated in platelet-derived growth factor and cytokines. Platelet-derived growth factor, transforming growth factor beta, vascular endothelial growth factor, and epidermal growth factor are among the substances that make up PRP. These substances are thought to encourage tissue growth and wound healing, and their administration is anticipated to encourage endometrial thickness in patients with a thin endometrium [6].

PRP therapy has not been associated with any negative side effects. No adverse responses are anticipated because the platelet-rich plasma is made from the woman’s blood. PRP injections might have varied effects on various women [10,11,12,13].

In vitro studies have shown that PRP is associated with increased stromal and mesenchymal cell proliferation, increased expression of regenerative enzymes, and enhancement in cell migration [9,10]. The regenerative ability of the endometrium in the normal menstrual cycle and throughout different surgical manoeuvres (e.g., curettage) or after birth is the basic principle of using the proliferation agents in the PRP [11]. 

A fertility clinic should run a blood test, an ultrasound, and a thorough assessment of the patient’s medical history to ascertain whether a patient is a candidate for ovarian rejuvenation using PRP. These are all common procedures in fertility testing. Once PRP has been delivered, the clinic will keep track of any changes in the patient’s hormone levels, perform another ultrasound to make sure the treatment is having the desired effect, and then determine when an IVF cycle should be performed.

Although the results are encouraging, its role in reproductive medicine is less established but intriguing. It could be hypothesized that the root cause of refractory endometrium is an endometrial injury, and so the infusion may promote endometrial regeneration, leading to increased EMT and thus endometrial receptivity, but with a debatable impact on clinical pregnancy and live birth.

This study aimed to determine if the PRP intrauterine infusion increases the EMT and improves the clinical pregnancy rate in patients with thin refractory endometrium, being one of the first studies to take an interest in this technique. Previous investigations that used PRP in endometrial pathology have found that PRP might improve endometrial receptivity, but the dosage and timing need to be carefully designed [9,10]. PRP infusion increased the endometrial thickness of patients with abnormal hysteroscopic findings but had no impact on the clinical pregnancy rate. The first study on the subject by Chang et al. (2015) found an increase in clinical pregnancy rate. Only one investigation studied the hysteroscopic injection of PRP in the sub-endometrial layer with promising results.

## 2. Materials and Methods

### 2.1. Study Design and Patients

This prospective single-arm self-control study was conducted in an IVF centre in Oradea, Romania during the period from March 2021 to July 2023. The study was conducted in accordance with the Declaration of Helsinki, and approved by the Ethics Committee of Pelican Clinical Hospital, Romania, no. 412/15.03.2021.

Patients with thin endometrium that met the following inclusion criteria were enrolled in this study. 

Inclusion criteria for patients: an IVF procedure resulting in at least one top-quality embryo, a signed accord for the procedure, hysteroscopy before embryo transfer with chronic endometritis diagnosed and treated if necessary, at least one cancelation history of embryo transfer without achieving 7 mm EMT after maximum oestradiol dose (9 × 2 mg daily) (ESTROFEM^®^, Novo Nordisk, Sydney, Australia), sildenafil (50 mg daily) (SILDENAFIL^®^, Actavis, Parsippany-Troy Hills, NJ, USA), pentoxifylline (400 mg daily) (Pentoxifilina SR^®^, Zentiva, Prague, Czechia), and low-dose aspirin (150 mg daily) (ASPENTER^®^, Terapia, Przeworsk, Poland).

Exclusion criteria for patients: pelvic cancer, acute inflammatory disease, intrauterine adhesions (Asherman syndrome), lack of consent, refusal of the hysteroscopic intervention, submucosal fibroma, or endometrial polyps. 

Endometrial thickness was measured with the vaginal probe at its thickest part in the longitudinal axis of the uterus with an ultrasound transvaginal probe of 5 to 9 MHz (Voluson E10, GE Healthcare, Chicago, IL, USA).

The control group included 51 patients that had undergone at least one attempt to transfer a good-quality blastocyst, but the EMT did not surpass 7 mm under hormonal therapy with adjuvant therapy (other than PRP).

The study group included the same 51 patients that had the embryo transfer performed under the oestradiol preparation protocol and intrauterine PRP infusion. 

The causes of infertility for the 51 patients were heterogeneous, such as: adenomyosis, demised ovarian reserve, tubal factor, and PCOS (polycystic ovarian syndrome). 

The aim of this study was to determine if platelet-rich plasma (PRP) intrauterine infusion increases endometrial thickness and improves clinical pregnancy rate.

Primary outcome: obtaining EMT of minimum 7 mm on the day of progesterone supplementation in the embryo transfer preparation after PRP infusion.

Secondary outcomes: clinical pregnancy rate, correlation between thin endometrium and chronic endometritis, and correlation between adenomyosis and thin endometrium. 

### 2.2. Endometrial Preparation

On the second day of the menstrual cycle after a transvaginal ultrasound evaluation, the patients started with a 2 mg dose of oestradiol, three times daily. After 7 days, another ultrasound evaluation for the EMT was carried out, and the oestradiol dosage increased to 6 tablets daily. The first uterine PRP infusion was performed under ultrasound guidance on the 7th day of endometrial preparation. After 2–3 days, the endometrial thickness was evaluated, and the oestradiol dosage increased to 9 tablets daily. The second PRP intrauterine infusion was performed on the 12th day of oestradiol administration, and the third PRP infusion was performed on the day of progesterone supplementation. On the day of progesterone supplementation, the level of progesterone needed to be under 1 ng/mL. The progesterone dosage was 1200 mg daily intravaginal administration (UTROGESTAN^®^ 200 mg Besins Healthcare) and 25 mg intramuscular daily (PROLUTEX^®^ 25 mg, Goodlife Pharma, Nairobi, Kenya). On the day of embryo transfer, the progesterone level was checked and a value over 8 ng/mL was required. The embryo transfer was performed under ultrasound guidance (trans-abdominally) with a Cook Medical’s Guardia™ Access Nano. After 10 days, beta HCG (human chorionic gonadotropin) was identified in maternal serum and the medication was continued with a transvaginal ultrasound at 4 weeks after the embryo transfer for clinical pregnancy confirmation.

### 2.3. PRP Preparation and Infusion

The blood was collected by venepuncture in an 8.6 mL tube containing 3.8% citrate solution, which was the anticoagulant from the kit (Endoret^®^ KIT 19). After the venepuncture, the tube was gently inverted 4 to 6 times. After a maximum of one hour after the venepuncture, the tube was centrifuged with Endoret^®^ (PRGF^®^) centrifuge System V in an 8 min cycle. The blood separated into 3 layers. The yellow layer is the plasma rich in growth factors, which contains platelets, the buffy coat with leukocytes is just above the red blood cells, and the third layer is the red blood cell layer on the bottom of the tube. The total volume of the plasma obtained after centrifugation depends on the haematocrit of the patient. The plasma transfer device was placed just below the surface of the plasma to be extracted, about 1–2 mm beneath the surface. Gently, the plasma fraction was aspired without the leukocytes or the erythrocytes in a 3 mL syringe. The plasma was activated by adding a precise quantity of Endoret activator (calcium chloride solution), meaning 0.02 mL/mL plasma. The content of the syringe was gently shaken. Activated plasma was kept at 37 °C for at least 40 min. Once the plasma had coagulated and retracted, the supernatant was filtered by applying pressure with the plunger. The obtained autologous activated platelet-rich plasma was infused in the uterine cavity under abdominal ultrasound guidance using a Wallace^®^ catheter for intrauterine insemination with the attachment of a syringe with 1ml of platelet-rich activated plasma. This was carried out on days 7, 12, and the day of progesterone supplementation (all cycles being for blastocysts, so day 5 embryo transfers). 

### 2.4. Statistical Analysis

Statistical analysis was performed using SPSS version 26.0 (IBM Corp, Armonk, NY, USA). The continuous variables are presented as means ± standard deviations for normally distributed data, and Student’s *t*-test was used to compare the differences before and after infusion with PRP.

## 3. Results

### 3.1. Characteristics of the Population

The average age of the group was 38.23 years old, with a deviation of 5.42 and a median of 38.36 (aged between 29 and 42 years old). For environmental characteristics, 17 patients were from urban areas, while only 6 were from rural areas and had body mass indices (BMI) between 18.0 and 41.0 kg/m^2^. These characteristics and infertility duration are shown in Table 1.

**Figure 1:**
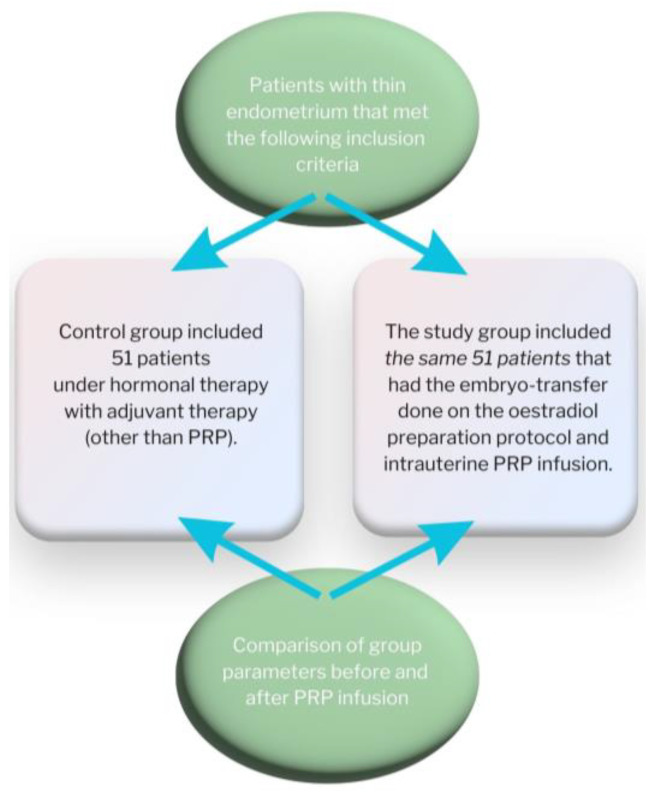
Work flow diagram.

The stages of the study are shown in Figure 1.

The parameters taken into account in the study were: thin endometrium, SET (single embryo transfer), testosterone, and clinical pregnancy after PRP treatment.

### 3.2. Parameters of the Group before the Infusion with PRP

The values of the parameters of the group before the infusion with PRP are given in Table 2.

The patients that had an embryo transfer preparation protocol without successful achievement of an endometrium thickness of minimum 7 mm had a mean value of 6.8 mm of endometrium thickness, one single embryo transfer, and no pregnancy.

### 3.3. Parameters of the Group after PRP Treatment

The values of the parameters of the group after PRP treatment are given in Table 3.

In the parameters following PRP, an increase in the size of the endometrium by 0.6 mm (from 6.8 mm to 7.41 mm) is observed. There is also an increase in clinical pregnancy. The study group and the control group had similar testosterone levels.

The endometrial thickness increased at 48–72 h after PRP infusion in all the patients and reached >8 mm on the day of progesterone administration. After application of PRP, the endometrial thickness was satisfactory in all the patients, who became pregnant.

### 3.4. Endometrial Thickness According to Age of Patients

For determining the effect of female age on the clinical pregnancy rate and the relationship with endometrial thickness, we divided patients into two different age groups: 18–35 and >35. It was observed that age is a determining factor. In patients over 35 years old, after the infusion with PRP, there was no significant increase in the thickness of the endometrium *p* > 0.01. In the case of patients younger than 35 years old, a real improvement in the thickness of the endometrium was observed (*p* < 0.01), Table 4.

The mean endometrial thickness decreased as a function of the patient’s age. The thickest endometrium was found in patients < 35 years of age and the thinnest endometrium was found in patients > 35 years of age.

### 3.5. Comparations between Group before and after PRP Treatment

By conventional criteria, this difference is considered to be statistically significant for all for all the parameters studied (*p* < 0.001), Table 5. This shows that the PRP treatment had a positive impact on the parameters that were followed for the evaluation of the success rate of the embryo transfer procedure. The most important ones were the endometrial thickness and the clinical pregnancy rate, which was statistically significant and was the main outcome of IVF.

## 4. Discussion

In IVF, the main factor alongside a good-quality embryo is endometrial thickness. The desired endometrial thickness of over 7 mm can be achieved either on a fresh or on a frozen embryo transfer under a preparation protocol. Despite all efforts, some patients do not achieve an endometrial thickness in different preparation cycles and should be counselled to proceed with the embryo transfer either way because they have a lower but acceptable live birth rate [12]. 

The limit of 7 mm of endometrial thickness has poor prognostic value for clinical pregnancy according to Yuan et al., but may seem to help with the implantation of the blastocyst [13]. 

Endometrial thickness, as the primary outcome measure in our study, was increased from 6.8 mm to 7.4 mm as a mean value, and this was also validated by studies that proved an increase in endometrial thickness by 0.6 mm compared to previous cycles of preparation [14,15]. Zadehmodarres et al., in their study, found that all patients showed sufficient endometrial growth after PRP intrauterine infusion, findings also seen in our study for all the patients that had a previous attempt at endometrial growth without PRP infusion [16]. The improvement of this parameter leads to lower cancellation rate and higher pregnancy and implantation rates. As an observation, even in patients that did not become pregnant, the endometrial thickness was higher. 

The adjuvant methods of endometrial preparation, such as low-dose aspirin, pentoxiphylline, low-molecular-weight heparin, sildenafil, and vaginal administration of oestrogens had no to little success in influencing endometrial thickness [16,17,18,19,20]. 

In comparison with other methods of endometrial growth stimulation, such as G-CSF (granulocyte-colony stimulating factor) the intrauterine infusion of PRP is more accessible for use. G-CSF administration via intrauterine infusion and during both the fresh and frozen embryo transfer cycles can improve the clinical pregnancy rate; however, whether G-CSF is effective in improving live birth rates is still uncertain and not a mainstream treatment [14,21,22,23,24]. 

PRP was first reported to have a significant impact on endometrial thickness in 2015 by Chang et al. in a study that showed an improvement in clinical pregnancy outcome for patients with thin endometrium. The study enrolled five patients with a history of thin endometrium, of whom four became pregnant after PRP infusion. This had a major impact in the field and started an interest in adjuvant therapies in embryo transfer preparation protocols [25].

PRP contains growth factors and cytokines that are considered to promote endometrial tissue healing and influence endometrial receptivity and embryo implantation and development [26]. After activation, the α-granules of platelets release a pool of biologically active proteins that stimulate cell proliferation, recruitment, growth, and differentiation [27]. According to the results of Anitua et al., activated PRP could improve the thickening of the endometrium as well as endometrial regeneration [27,28].

In the tubal factor group, there is a higher chance of ectopic pregnancy, and this is associated with a thin endometrium. The endometrium in PCOS women has been observed to overexpress androgen receptors and fail to downregulate oestrogen receptor-α during secretion, leading to an abnormal endometrial phenotype and function [28,29].

The need for standardisation in the PRP preparation technique could help develop more studies and find the implication of this adjuvant therapy in assisted reproduction. The preparation protocol of the activated PRP or PRP is different in almost every study found and can lead to very different results. The fact that each patient is unique and has a different blood cell count means that in every patient the final product of PRP is different and will act in different ways because of different concentrations. Leukocytes, for example, have a definitory role in the outcome and should be taken into consideration. Yanna Ban et al.’s study showed that leukocyte-poor platelet-rich plasma (LP-PRP) treatment could improve clinical pregnancy rate and live birth rate in recurrent-implantation-failure patients undergoing frozen embryo transfer (FET) cycles [30].

The number of platelets is also dependent on the probe and the preparation protocol used and could have different results, and therefore should be standardized. The platelets from the autologous plasma activate cytokines with anti-inflammatory properties that regenerate tissues like IL-10 [30]. The quantity of PRP infused is also a parameter that can vary. According to Tehraninejad et al., a dose of 1 mL showed a better effect compared to a dose of <0.5 mL on the clinical pregnancy rate [31,32,33,34]. We infused 1 mL for each dose and found good results in the study group, although we did not try to infuse less in any of the patients. 

The frequency of administration was three times during the preparation of the embryo transfer cycle as follows: days 7, 12, and on the day of progesterone supplementation, meaning three doses of PRP intrauterine infusion. Dogra Y et al. used a similar frequency of administration of PRP intrauterine infusion with three doses starting from day 8 of preparation protocol every 48 h until an 7 mm endometrial thickness was achieved, and a clinical pregnancy rate of 25% was achieved after the procedure [35].

The majority of studies use the intrauterine infusion of activated PRP similarly to our technique to maximize the effect of an endometrial preparation with high oestrogen doses and without satisfactory results. The activator is applied in a dose according to the manufacturer and usually is a product of thrombin powder and calcium chloride. Another technique is the endometrial injection of PRP under hysteroscopic guidance via an endoscopic needle, although few studies have been conducted in this regard and validation is required in comparison to the infusion technique. PRP injections and injections of the endometrial cells suspended in PRP into the endometrium enhanced cell proliferation and angiogenesis according to Efendieva et al. [36]. PRP injection into the sub-endometrial region is consistent with the histologically proven regeneration of the endometrium. Agarwal et al. observed an improvement in endometrial thickness and higher clinical pregnancy rates in their study using hysteroscopic sub-endometrial PRP injection [37].

PRP intrauterine infusions could possibly help with endometrial receptivity and studies have started to study this with encouraging results [4,37,38]. It is hypothesised that the endometrial stem cells are stimulated by the regenerative properties of PRP and improve endometrial response and embryo implantation [39].

In both natural clinical pregnancy and IVF cycles, the presence of endometrial scarring and chronic inflammation can make embryo implantation difficult. The application of PRP infusions attempts to reduce inflammation and enhance progesterone receptor activation, which stimulates the endometrium’s healthy growth [35,36].

PRP plays an important role in the proliferation of cytokines that are released in the inflammation process and proliferation of epithelial cells and endometrial mesenchymal stem cells. Among these, VEGF is important in the vascularization process. By causing the increased expression of molecules that attract stem cells and increased cell migration, PRP treatment can stimulate endometrial receptivity as well as growth [40].

One of the primary endometrial markers of receptivity, HOXA 10, was found in a high level in patients with PRP uterine infusion. In the process of the autologous PRP administration, there were no side effects. In the balance of cost-effectiveness, PRP tends to win due to encouraging results and easy accessibility [41,42,43].

In our study group, the results were encouraging, all patients with a certified history of “thin endometrium” showed endometrial growth, and the procedure had a significant clinical pregnancy rate compared to the control group. 

Limitations of the study: a small sample size, the lack of multicentric validation, and the small number of studies conducted on the topic of thin endometrium due to the low prevalence in IVF. 

In terms of other new and interesting therapies, we highlight the stem cell therapy and the endometrial receptivity test (ERA test) for personalized treatment in refractory cases of thin endometrium. The human umbilical cord-PRP treatment can restore the injured endometrium and is a suitable candidate for therapeutic management of patients with endometrial pathologies using stem cells. The findings of this study align with previous research suggesting that PRP intrauterine infusion may positively impact endometrial thickness in IVF. The growth factors present in PRP likely contribute to increased angiogenesis, cellular proliferation, and improved tissue remodelling, resulting in a thicker and more receptive endometrium. However, further research is needed to elucidate the underlying mechanisms and establish optimal PRP treatment protocols.

Many patients are still experiencing recurring implantation failure (RIF), even though there are significant advancements in embryo election and quality enhancement.

Implantation failure is seen in a subgroup of patients going through IVF who may also benefit from PRP treatment along with the thin endometrium group (which can also be found in recurrent implantation failure). PRP administration seems to improve implantation, clinical pregnancy, chemical pregnancy, on-going pregnancy, live birth rates, and endometrial thickness in women with previous implantation failure according to an encouraging meta-analysis [44]. The lack of evidence-based treatments for poor endometrial receptivity has become an impediment issue in the ART industry. Intrauterine adhesions are a major complication that will cause thin endometrium. In new studies, the use of the PRP treatment after hysteroscopic adhesiolysis could decrease the intrauterine adhesion forming and improve the menstrual cycle and hypothetically the endometrial thickness and receptivity [45,46,47,48]. Researchers and clinicians have encountered difficulty in creating a potent therapeutic approach. In recent years, increasing evidence has demonstrated the beneficial effects of autologous platelet-rich plasma (PRP) in the treatment of endometriosis.

## 5. Conclusions

PRP has a positive effect on promoting endometrial proliferation, improving embryo implantation rate and clinical pregnancy rate for women with thin endometrium. The observed improvements in endometrial thickness following PRP treatment indicate its ability to positively influence endometrial receptivity, ultimately leading to improved IVF outcomes. Continued research and controlled clinical trials are necessary to further explore the full potential of PRP in reproductive medicine and establish standardized protocols for its application in IVF treatment.

## Figures and Tables

**Table 1:** Baseline characteristics of the group.

Baseline Characteristics of the Group	Group MD ± SD
Age of patients (years)	38.23 ± 4.42
Environment	
Urban	23 (45.09%)
Rural	28 (54.91%)
BMI (kg/m^2^)	26.1 ± 3.7
Infertility duration (years)	5.8 ± 2.7

**Table 2:** Parameters of the group before the infusion with PRP.

Parameters	MD ± SD
Thin endometrium (mm)	6.7 ± 1.32
SET (single embryo transfer)	1 ± 1.18
Testosterone (ng/mL)	0.495 ± 0.62
Pregnancy before PRG treatment	0 ± 0.38

**Table 3:** Parameters of the group after PRP treatment.

Parameters	MD ± SD
Thin endometrium (mm)	7.81 ± 0.71
SET (single embryo transfer)	2 ± 1.08
Testosterone (ng/ML)	0.797 ± 0.92
Pregnancy after PRG treatment	0.5 ± 0.12

**Table 4:** Endometrial thickness according to age of patients.

	1–7 mm		7.1–12 mm		*p* Value
	Control Group		Study Group (the Same Group after PRP Treatment)		
Age	n	%	n	%	
≤35	32	62.74%	31	60.78%	0.0001
>35	19	37.26%	17	33.33%	0.029

**Table 5:** Comparations of parameters of the group after PRP treatment.

Parameters	MD ± SD	*p* Value
Thin endometrium (mm)	7.25 ± 1.015	0.0001
SET (single embryo transfer)	1.5 ± 1.13	0.0019
Pregnancy after PRG treatment	0.25 ± 0.2	0.0004
